# Electrodeposition of BiVO_4_ Nanoparticles on TiO_2_ Nanotubes: Characterization and Synergetic Photocatalytic Degradation Activity of Amido Black Dye

**DOI:** 10.3390/molecules30214283

**Published:** 2025-11-04

**Authors:** Kawther Ben Mabrouk, Syrine Sassi, Ines Khemissi, Rabia Benabderrahmane Zaghouani, Lotfi Khezami, Hamza Elfil, Amal Bouich, Bernabé Mari Soucase, Anouar Hajjaji

**Affiliations:** 1 Desalination and Natural Water Valorization Laboratory (LaDVEN), Water Research and Technologies Center (CERTE), P.O. Box 273, Soliman 8020, Tunisia; 2 Photovoltaic Laboratory, Research and Technology Centre of Energy (CRTEn), P.O. Box 95, Hammam-Lif 2050, Tunisia; 3 School of Design Engineering, Departamento de Fisica Aplicada, Universitat Politecnica de Valencia, Cami de Vera, 46022 Valencia, Spain; 4 Chemistry Department, College of Science, Imam Mohammad Ibn Saud Islamic University (IMSIU), Riyadh 11623, Saudi Arabia; 5 Laboratory of Advanced Materials and Process Engineering, Faculty of Sciences, Ibn Tofail University, Kenitra 14000, Morocco

**Keywords:** TiO_2_ nanotubes, BiVO_4_ nanoparticles, heterojunction, photocatalysis, amido black

## Abstract

To enhance the photocatalytic performance of TiO_2_ nanotubes (NTs) for the degradation of Amido Black as an organic pollutant, electrodeposition of bismuth vanadate (BiVO_4_) nanostructures was successfully applied. The effect of electrodeposited BiVO_4_ (25 s, 50 s, 150 s, 250 s), followed by a thermal treatment on TiO_2_-NTs, was studied. The structures of the as-prepared samples were characterized by X-ray diffraction (XRD). Morphological behavior was investigated using Scanning Electron Microscopy (SEM) and Transmission Electron Microscopy (TEM), both coupled with EDX. Optical characterizations were performed using photoluminescence and diffuse reflectance spectroscopy. The BiVO_4_/TiO_2_ NTs sample with 50 s deposition time gave the highest photocatalytic performance for Amido Black degradation, 99.4% after 150 min under UV light. This result has been achieved due to the structure and the optical properties of the sample. The heterojunction of both catalysts showed the synergetic effect on the photocatalytic performance where they remained stable after five cycling runs. Furthermore, quenching tests were conducted and proved that superoxide radicals (O_2_^•^) are the main active species during photodegradation process.

## 1. Introduction

Dyes have been in widespread use in a wide range of industries for decades. Uncontrolled discharge of untreated hazardous dyes into freshwater causes harmful impacts on human health and ecosystems. Therefore, the mineralization of dyes in aquatic systems has become a top priority due to its negative effects. One of the most widely used dyes in clothing, beauty, food, printing, and even the pharmaceutical industry is Amido Black [1,2,3]. In order to achieve the complete degradation of such a pollutant in water, several technologies have been applied [4,5,6,7,8]. The most reputable and efficient techniques for organic materials removal are Advanced Oxidation Processes (AOPs). The AOP’s methods are designed for the removal of organic materials by way of reaction with reactive oxygen species (ROS), such as superoxide free radicals (O_2_^•^)^−^ and hydroxyl radicals (HO^•^), which are highly oxidative to the point of utter degradation or mineralization of the mentioned pollutants [9,10]. As these methods continue to evolve throughout the years and diversify in terms of energy sources for the generation of free ROS in aqueous solution, including sonocatalysis, electrocatalysis, photocatalysis, and piezocatalysis [11,12,13,14], the catalysts also evolve in terms of type, state, morphology, and size. One of the most exploited photocatalysts is TiO_2_, with its excellent properties and its band edge positioned for adequate potential for radical generation [15,16]. The variation in morphology of TiO_2_ expressed different results for the photodegradation of organic pollutants. The techniques for the TiO_2_ nanotubes synthesization varied as many emerged, such as the hydrothermal method [17], the template method [18] and the commonly used the anodization method [19], which is a simple zero-cost method that consists of immersing the electrodes in electro-catalytic solution for 2 h at 60 V; according to the literature, the obtained morphology is highly ordered and vertically oriented. Due to these characteristics, the nanotubular morphology possesses a high specific surface area, a fast electron transfers and great photocatalytic activity [20,21,22]. Even though the TiO_2_ nanotubes exhibit potential properties, they possess some limitations towards their effectiveness and yield, as this material presents a high recombination rate and limited spectrum performance [23,24]. To overcome these obstacles, a modification of the band alignment is proposed by coupling TiO_2_ with other photocatalysts, such as Cu_2_O, PbS, ZnO, NiO, BiVO_4_ and Fe_2_O_4_ [25,26,27,28,29,30,31].

For instance, for the degradation of Methylene Blue, Shaban et al. [32] synthesized TiO_2_ nanoribbons and obtained a photocatalytic degradation of 97.5% for 180 min under UV light, while Alias et al. [33] prepared TiO_2_ nanoflowers and achieved a photodegradation of 98.95% for 180 min under solar light. Dong et al. [34] also reached a 100% degradation of Methylene Blue in only 40 min under UV light with hierarchical mesoporous TiO_2_ nanoshell. However, TiO_2_ nanotubes are considered one of the best morphologies and are highly adequate for photocatalytic application [35,36,37], as Lin et al. [38] reported the difference between TiO_2_ nanoparticles and TiO_2_ nanotubes for the degradation of Methylene Blue, where the nanotubular morphology excelled with a removal efficiency of 99% in 25 min under UV light. Recently, Hajjaji M.A. et al. [39] demonstrated that deposition of Pt on to the surface of TiO_2_ nanotubes contributes to the improvement in photocatalytic activity of Black Amido degradation compared to the pure sample. The authors reached the highest photocatalytic performance (97% degradation) after 90 min under UV irradiation.

The following Table 1 is a comparison between some results related to the use of catalysts/TiO_2_ for the degradation of Amido Black as an organic dye. BiVO_4_ has proven itself to be an excellent photocatalyst due to its narrow band gap and potential properties; Missaoui et al. [31] prepared nanoflower-like BiVO_4_ nanostructured films for peroxymonosulfate (PMS) activation for the degradation of Rhodamine B (RhB) in aqueous solution. Under optimized conditions, they achieved total degradation in 17 min. Also, Nhat et al. [40] decorated BiVO_4_ pine-like morphology for the photodegradation of Sulfamethoxazole; they reached a degradation of 98.8% under 210 min irradiation. The adequate band edge position of BiVO_4_ and its excellent photocatalytic properties makes it a potential candidate for a heterojunction with TiO_2_ nanotubes; this will be of interest to reducing the recombination by separating the pairs’ electron/holes, thus enhancing the photocatalytic performances. For instance, Saidurga et al. [41] demonstrate the remarkable efficiency of hydrothermally synthesized BiVO_4_–TiO_2_ nanocomposites for the degradation of Rhodamine B. With a dye concentration of 5 × 10^−5^ M and a catalyst amount of 2 g/L, the 1:3 BiVO_4_–TiO_2_ composite achieved 97.1% degradation in 60 min under sunlight, with stability maintained over five cycles. Also, the mechanism analysis reveals that hydroxyl radicals are the main active species.

In this work, an effective approach to fabricating Z-scheme photocatalysts and a heterojunction of BiVO_4_/TiO_2_ nanotubes will be synthesized with the electrodeposition method at different conditions to study the effect of the deposition on the properties of TiO_2_ nanotubes, and photocatalytic activity will be investigated for the degradation of Black Amido.

## 2. Results

### 2.1. Structural Analysis

#### 2.1.1. XRD Analysis

**Figure 1:**
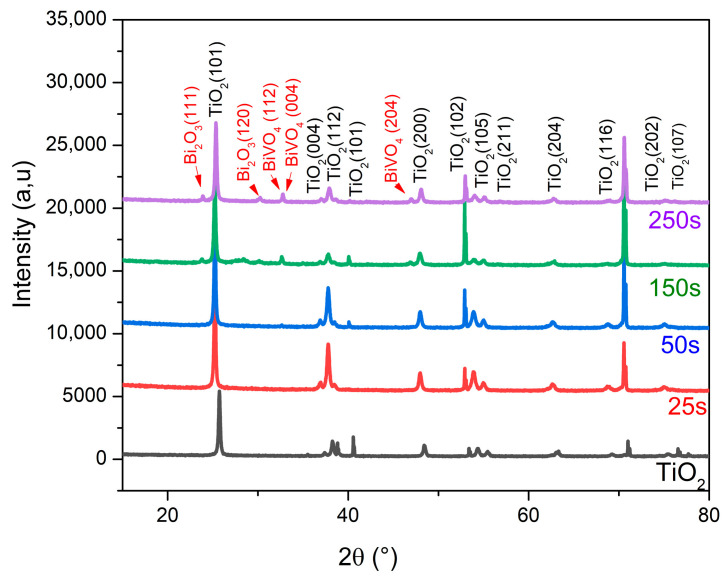
XRD patterns of BiVO_4_/TiO_2_ at different electrodeposition times (25, 50, 150, 250 s).

To investigate the structural properties and crystallinity of bare TiO_2_ nanotubes and BiVO_4_/TiO_2_ nanocomposites at different electrodeposition times of 25, 50, 150 and 250 s, X-ray diffraction was performed. Figure 1 shows the XRD patterns of each sample, where they all show a preferred orientation with high crystallinity corresponding to TiO_2_ anatase (101) at 2θ = 25.7°; these samples also show other orientations for the anatase phase at different angles (112), (004), (200), (105), (211), (204), (116), (202) and (107), corresponding to JCPDS card no. 21-1272. After the deposition of BiVO_4_ on the surface of the TiO_2_ nanotubes, one may notice the appearance of small, sharp peaks at 2θ = 29.6° and 32° related to the (112) and (004) monoclinic scheelite BiVO_4_ phase (JCPDS card No. 75–1866). One can observe the presence of narrow XRD peaks corresponding to (111) and (120) Bi_2_O_3_ according to the reference JCPDS card No. 76-1730. These BiVO_4_ and Bi_2_O_3_ peaks are only visible for deposition times at 150 s and 250 s; it seems that for the lower deposition times, the peaks are unlikely to be detected, which could be due to their small size compared to the higher deposition time.

**Figure 2:**
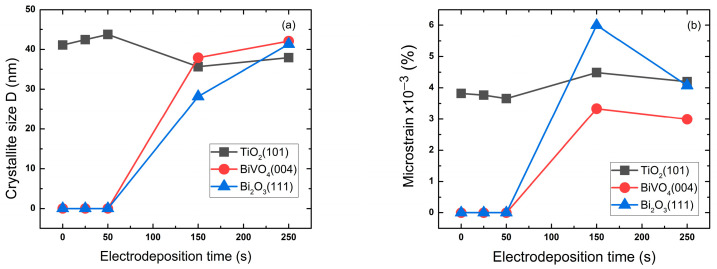
Crystallite size (**a**) and microstrain (**b**) of BiVO_4_/TiO_2_ at different electrodeposition times: 25 s, 50 s, 150 s, 250 s.

Figure 2 shows the variation in crystallite size and microstrain with the variation in Bi deposition time of the preferred orientation for each composite: TiO_2_ (101), BiVO_4_ (004) and Bi_2_O_3_ (111). These variations were estimated through the following equations [44].

Debye–Scherer formula:

(1) $$D=\frac{K\lambda }{\beta \cos\theta }$$

Wilson equations:

(2) $$\varepsilon =\frac{\beta }{4tan(\theta )}$$

where *K* is the shape factor, *λ* the X-ray wavelength, *β* the full width at half maximum of the peak, and *θ* the Bragg angle.

The exhibited values of the crystallite size corresponding to TiO_2_ (101) in Table 2 increase as the Bi deposition time increases from 0 to 50 s to reach 43.7 nm, then unexpectedly decreases for 150 and 250 s. This could be in correlation with the formation mechanism of the Bi layers. However, the crystallite size of BiVO_4_ and Bi_2_O_3_, as shown in Table 2, continues to increase with the deposition time to a maximum of 42 and 41.3 nm, respectively. The correlation between crystallite size and microstrain [45] is distinguishable for the BiVO_4_, where the microstrain decreases with increasing crystallite size and electrodeposition time, which means the structure is stabilizing with less defects and dislocations. For Bi_2_O_3_, it appears that this structure has the highest microstrain, which could be due to the high defects present in the structure and low crystallinity. However, the 50 s sample TiO_2_ (101) has the largest crystallite size and the lowest microstrain; this could improve charge transport efficiency with fewer grain boundaries for charge carriers to overcome [46].

#### 2.1.2. XPS Analysis

**Figure 3:**
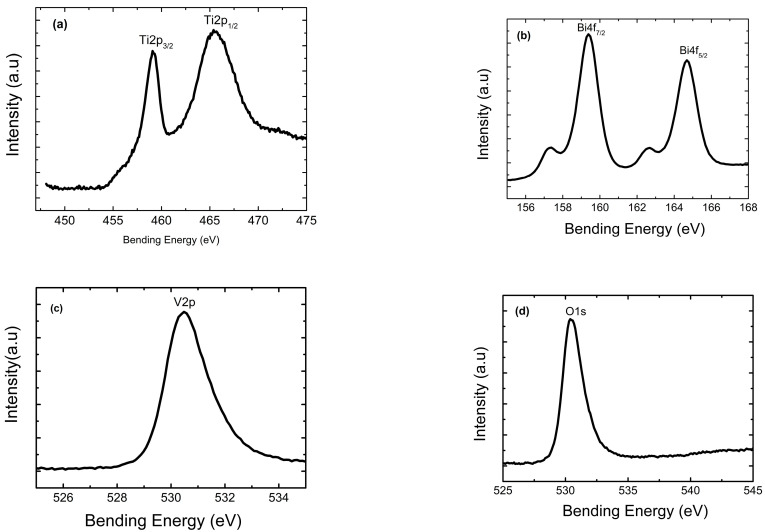
High-resolution XPS spectra of the BiVO_4_/NTs-TiO_2_ 50 s composite film: (**a**) Ti 2p, (**b**) Bi 4f, (**c**) V 2p, and (**d**) O 1s.

The high-resolution XPS spectra of Ti 2p, Bi 4f, V 2p and O 1s for the BiVO_4_/NTs-TiO_2_ nanocomposite are shown in Figure 3, implying the existence of the elements Ti, Bi, V and O in the nanocomposite film. The strong peaks at 459.1 eV and 465.4 eV were assigned to Ti 2p_3/2_ and Ti 2p_1/2_ (Figure 3a), respectively, and the energy difference between them was 6.3 eV, matching well with the data of Ti^4+^ in TiO_2_ [47,48]. The peaks appearing at 159.4 eV and 164.7 eV were attributed to Bi 4f_7/2_ and Bi 4f_5/2_ (Figure 3b), respectively, and the peaks at 530.5 eV originated from V 2p (Figure 3c). The above XPS results confirmed that the valence states of Bi and V were Bi^3+^ and V^5+^ in the composite film, respectively [47,48]. The characteristic peak of O 1s in Figure 3d at 530.0 eV is attributed to oxygen. Combining the results from XRD and XPS analyses, we concluded that the monoclinic BiVO_4_-modified TiO_2_ nanotube nanocomposite film has been successfully prepared through the anodic oxidation and different electrodeposition times.

### 2.2. Morphological Analysis

#### 2.2.1. SEM Analysis

**Figure 4:**
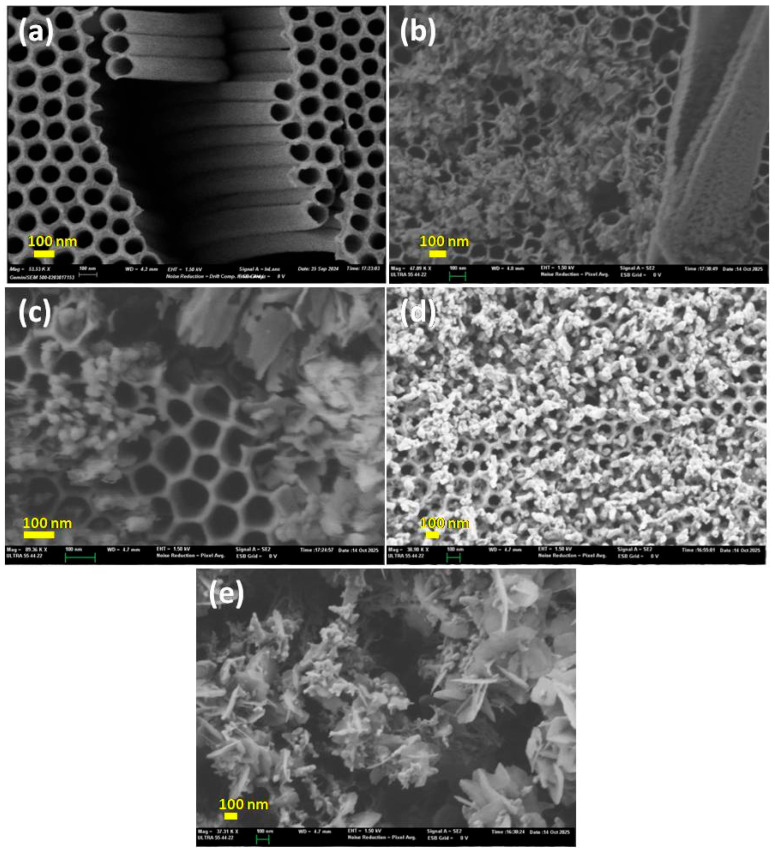
SEM imaging of (**a**) pure TiO_2_ nanotubes and BiVO_4_/TiO_2_ at different electrodeposition times (**b**) 25 s, (**c**) 50 s, (**d**) 150 s, (**e**) 250 s.

To investigate the effect of the deposition time on morphology of the samples, Scanning Electron Microscopy was conducted for pure TiO_2_ nanotubes and BiVO_4_/TiO_2_ nanotubes composites at different Bi deposition time 25 s, 50 s, 150 s and 250 s. The SEM images were taken at different magnifications to emphasize complementary structural features of the BiVO_4_/TiO_2_ samples. While it was not possible to acquire all images under identical magnification, the scale bars are provided to allow accurate comparison. This approach ensures that both the general morphology and the nanoscale features are clearly visible. Figure 4a shows the formation of TiO_2_ in regular and vertically ordered tubes on the surface of Ti substrate; after the deposition of BiVO_4_, irregularly shaped particles started to appear at 25 s deposition time (Figure 4b) and the nanosheets of BiVO_4_ were dispersed unevenly and agglomerated at some points; with the increase in deposition time to 50 s (Figure 4c), the size and number of the nanosheets increased. These nanosheets grew in size and entangled each other to almost form porous, nanoflower-like structures covering the TiO_2_ nanotubes at higher Bi deposition time (Figure 4d,e), where the nucleation kinetics change, while Bi nanoparticles nucleate and various BiVO_4_ morphologies emerge [49].

**Figure 5:**
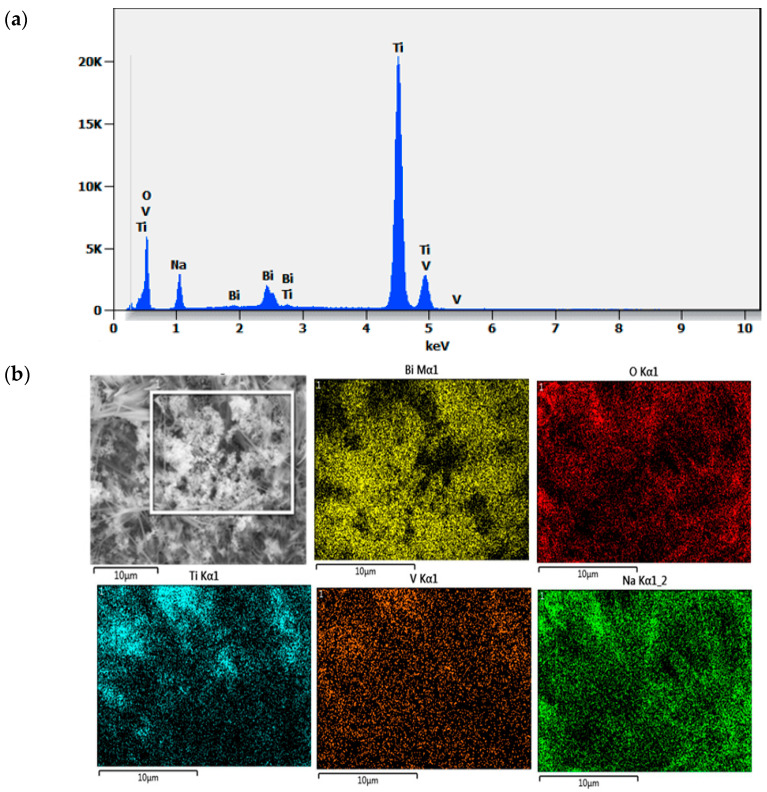
(**a**) EDX spectra of BiVO_4_/TiO_2_ at 250 s electrodeposition time, (**b**) mapping image of the elements.

For a more in-depth study, coupled SEM characterization and EDX were conducted to identify the elements and the purity of the samples. Figure 5a shows the EDX spectra where peaks of Ti, O, V, and Bi are extremely visible for the 250 s BiVO_4_/TiO_2_ sample; Figure 5b shows the mapping images for the element where the vanadium and bismuth are largely distributed on the surface, which confirms the successful deposition of BiVO_4_ nanoparticles on the surface of TiO_2_ nanotubes. Table 3 exhibits the weight percentage of each element. It is distinguishable that Na has about 10% of total wt%; this could be due to the final 30 min wash of the samples with 1 M NaOH to eliminate the yellow-green residual V_2_O_5_ excess layer.

#### 2.2.2. TEM Analysis

**Figure 6:**
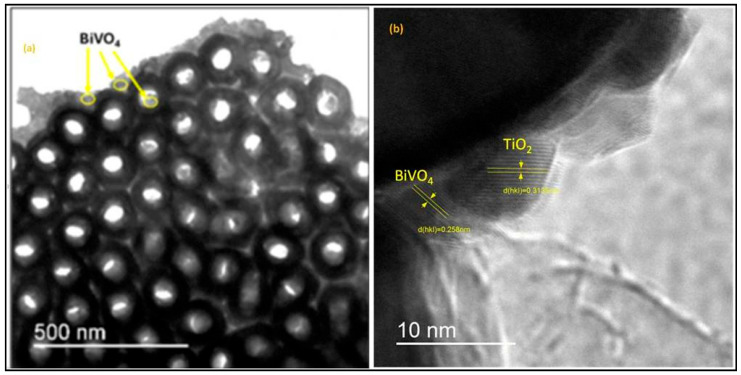
(**a**) Bottom view TEM image of TiO_2_ nanotubes decorated with BiVO_4_ nanoparticles at 50 s electrodeposition time, (**b**) HRTEM imaging of the BiVO_4_/TiO_2_ interface.

The Transmission Electron Microscopy investigation shows the TEM Images of BiVO_4_ nanoparticles deposited on TiO_2_ nanotubes at 50 s electrodeposition time; Figure 6a is bottom view image of the nanotubes with a diameter of 150 nm and an internal diameter of 68 nm. It is clearly visible that the BiVO_4_ nanoparticles are attached to the external and internal membrane of the nanotubes. Figure 6b is a high-resolution TEM image of the BiVO_4_/TiO_2_ interface. The HRTEM micrograph in Figure 6b, with its sharply resolved lattice fringes, clearly confirms the formation of this crucial BiVO_4_/TiO_2_ heterojunction. Careful analysis of the fringes shows two key interplanar spacings: the d(hkl) = 0.258 nm corresponding to the (200) plane of monoclinic BiVO_4_ (JCPDS No. 75-1866), while the d(hkl) = 0.313 nm is indexed to a specific lattice plane of TiO_2_ anatase (JCPDS No. 21-1272). This intimate physical contact is essential for our composite’s functional performance, as it ensures efficient charge transfer and the successful separation of photogenerated electron–hole pairs, which is a key mechanism for enhancing our photocatalytic system.

**Figure 7:**
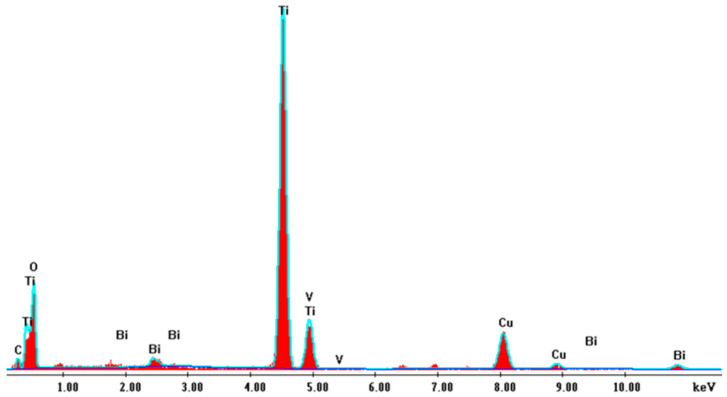
EDX spectra of BiVO_4_/TiO_2_ at 50 s electrodeposition time.

The performed EDX in Figure 7 confirms the high purity of the sample and the evidence of existing elements Ti, O, Bi, V.

### 2.3. Optical Analysis

#### 2.3.1. Photoluminescence

**Figure 8:**
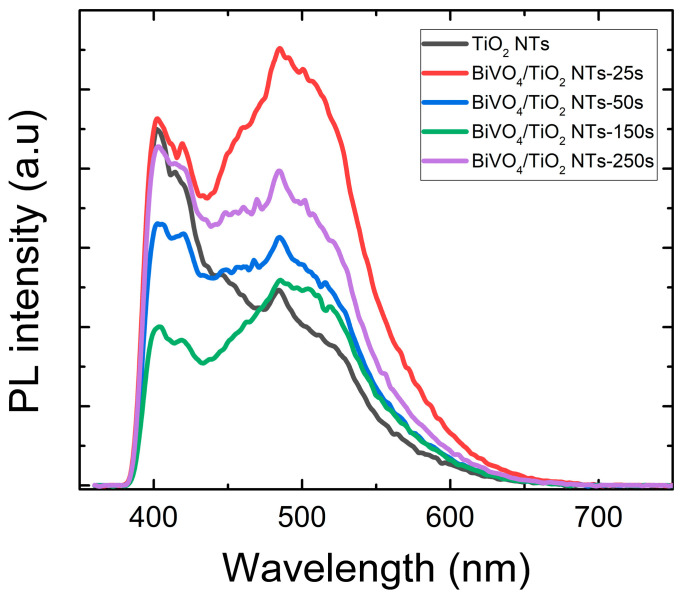
Photoluminescence spectra of bare TiO_2_ and BiVO_4_/TiO_2_ nanotubes with different electrodeposition times: 25 s, 50 s, 150 s, 250 s.

The migration, transfer and trapping of the photogenerated charge carrier’s electron–hole pairs in TiO_2_ were investigated with photoluminescence assessment, which also apprises the defects present in the band gap [50]. Figure 8 depicts the photoluminescence spectra of the BiVO_4_/TiO_2_ samples at different electrodeposition time 25 s, 50 s, 150 s and 250 s to better understand the impact of BiVO_4_ depositions on the optical properties of TiO_2_. Compared to the bare TiO_2_ nanotubes, it seems that all the samples present the same peaks located between 390 nm and 420 nm corresponding to 3~3.2 eV, which is compatible with the electronic transition between conduction and valence bands. The collection of peaks detected between 450 nm and 550 nm is related to the oxygen vacancies in TiO_2_ structure. Since the recombination of electron/hole is derived from the PL emission, the weaker the PL intensity, the lower the recombination rate.

The detected data reveal a non-monotonic trend in charge carrier recombination. Compared to the bare TiO_2_ nanotubes, the 50 s and 150 s samples exhibit a significant decrease in PL intensity. This indicates a lower electron–hole recombination rate, which we attribute to the successful formation of a BiVO_4_/TiO_2_ heterojunction at these deposition times. The heterojunction promotes the spatial separation of photogenerated charges, thereby suppressing their recombination. In contrast, the 25 s and 250 s samples show a higher PL intensity than pure TiO_2_, signaling enhanced recombination. For the 25 s sample, the low BiVO_4_ coverage is likely insufficient for optimal heterojunction formation; instead, the deposited material may introduce defective states that act as recombination centers. For the 250 s sample, the excessively thick and aggregated BiVO_4_ layer (as seen in SEM) introduces a high density of grain boundaries and defects, hindering charge transfer and promoting non-radiative recombination within the overlayer itself. This trend confirms that an optimal BiVO_4_ deposition time is critical to maximizing charge separation, with the 50 s sample achieving the most effective balance [51].

#### 2.3.2. Diffuse Reflectivity

**Figure 9:**
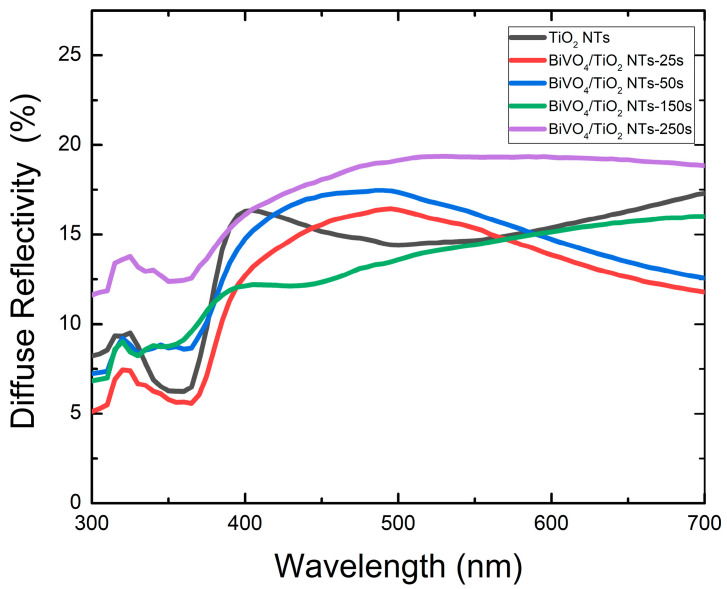
Diffuse reflectivity spectra of bare TiO_2_ and BiVO_4_/TiO_2_ nanotubes with different electrodeposition times: 25 s, 50 s, 150 s, 250 s.

The diffuse reflectivity spectra of as-fabricated samples have been investigated with UV–Vis spectroscopy as shown in Figure 9; the spectra depict a minimum for all samples up to 380 nm, which corresponds to the gap energy of anatase TiO_2_ 3.2 eV; then, the diffuse reflectivity increases for higher wavelengths. It seems that 250 s electrodeposition time of BiVO_4_ nanoparticles presents the highest reflectivity, which could be due to the surface characteristics; however, on the contrary, the 25 s sample showed a lower reflectivity in comparison to pure TiO_2_ and other samples. To determine the band gap of each sample, the Kubelka–Munk formula was used to create the Tauc plots (F(R)*hʋ*)^1/2^ vs. *h*ʋ [52].

(3) $$F\left(R\right)=\frac{{\left(1-R\right)}^{2}}{2R}$$

After the exploitation of the Tauc plots, the collected optical band gap energy of each sample is presented in Table 4. The bare TiO_2_ nanotube presented band gap energy of 3.3 eV, which is almost consistent with the literature, in that the deposition time has an intense effect on the optical band gap energy. The band gap decreased to a minimum of 2.6 eV for the 50 s sample, which possesses the largest TiO_2_ crystallite size and lowest microstrain, indicating high crystallinity. However, for longer deposition times (150 s, 250 s), it increased to 2.66 eV and 2.7 eV, respectively. This reversal coincides with a significant reduction in the apparent TiO_2_ crystallite size (from 43.74 nm to 35.64 nm) and a probable increase in microstrain (Figure 2b). We attribute this not to a contradiction with the XRD results, but to the effects of thermal treatment on the composite. At high Bi loadings, the vigorous interfacial reaction and stress during the formation of a thick BiVO_4_ layer introduce lattice defects and strain into the TiO_2_ substrate. This disrupts the long-range order, broadening the XRD peaks and reducing the calculated crystallite size. The associated increase in band gap is consistent with the quantum confinement effect induced by this effective reduction in crystalline domain size and the rise in defect density within the TiO_2_ structure [49,50].

The size of the crystal plays an important role in determining the band gap of the semiconductor, with smaller sizes leading to larger band gaps due to quantum confinement effects, where small particle size leads to discrete energy levels and band splitting [53]. Furthermore, the effect of crystal size on the band gap shift is found to be parallel to that of intrinsic strain [54].

### 2.4. Photodegradation of Black Amido with BiVO_4_/TiO_2_

**Figure 10:**
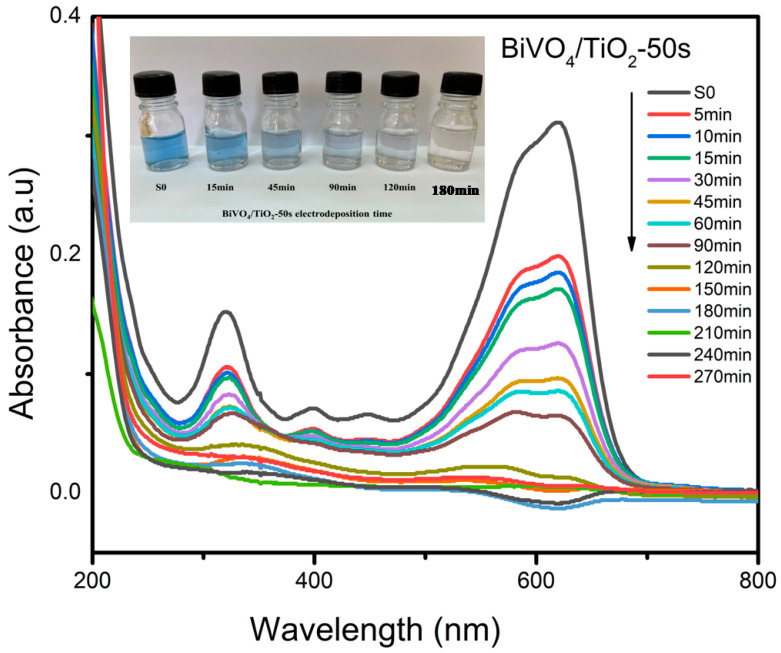
The absorbance of Amido Black after photodegradation for bare TiO_2_ and BiVO_4_/TiO_2_ nanocomposites at 50 s electrodeposition time.

The photodegradation of Black Amido was conducted under UV light at different times (5, 10, 15, 30, 45, 60, 90, 120, 150, 180, 210, 240 and 270 min) and with bare TiO_2_ nanotubes and BiVO_4_-NPs/TiO_2_-NTs as photocatalysts with different electrodeposition times 25 s, 50 s, 150 s, and 250 s. Black Amido was chosen as an organic pollutant due to its complex chemical structure; the evaluation of the concentration during the photocatalysis process was assessed with UV–Vis absorption studies using the Beer–Lambert relation at a maximum wavelength of λ= 618 nm. Figure 10 depicts the absorbance spectra after the photocatalytic activity of Amido Black AB. The inset image shows how the blue color of AB is gradually decreasing in intensity as the irradiation time increases, and is completely gone at 180 min.

Following Equation (4), the removal % of AB with BiVO_4_/TiO_2_ photocatalyst was determined [55].

(4) $$\mathrm{Removal}\;\mathrm{of}\;\mathrm{dye}\;(\%)=(1-\frac{C}{C_{0}})\times 100$$

**Figure 11:**
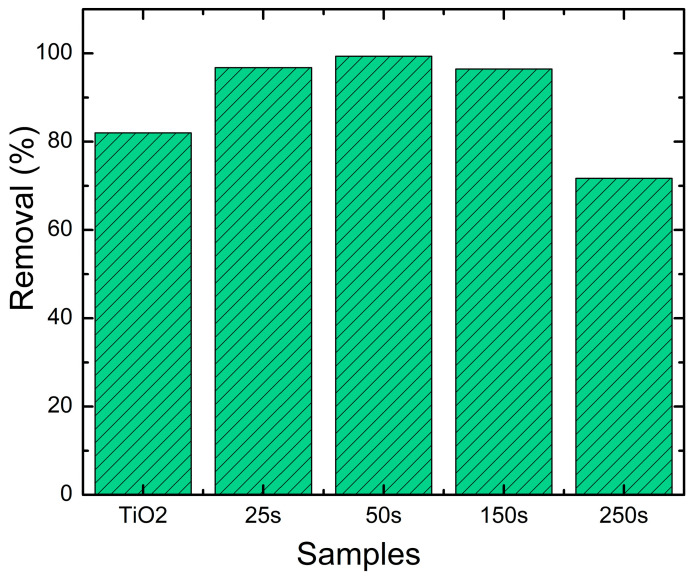
Photodegradation efficiency of bare TiO_2_ and BiVO_4_/TiO_2_ nanocomposites with different electrodeposition times: 25 s, 50 s, 150 s, 250 s for Amido Black removal.

It seems that the sample BiVO_4_/TiO_2_-50 s electrodeposition time reached a total degradation of Black Amido 99.4% at 150 min under UV light as illustrated in Figure 11, which is considered the highest compared to the other samples; this result agrees with the characterization results and the addition of BiVO_4_ to favor the production of reactive oxygen species. The active sites responsible for the degradation of the pollutant increase with the presence of BiVO_4_.

**Figure 12:**
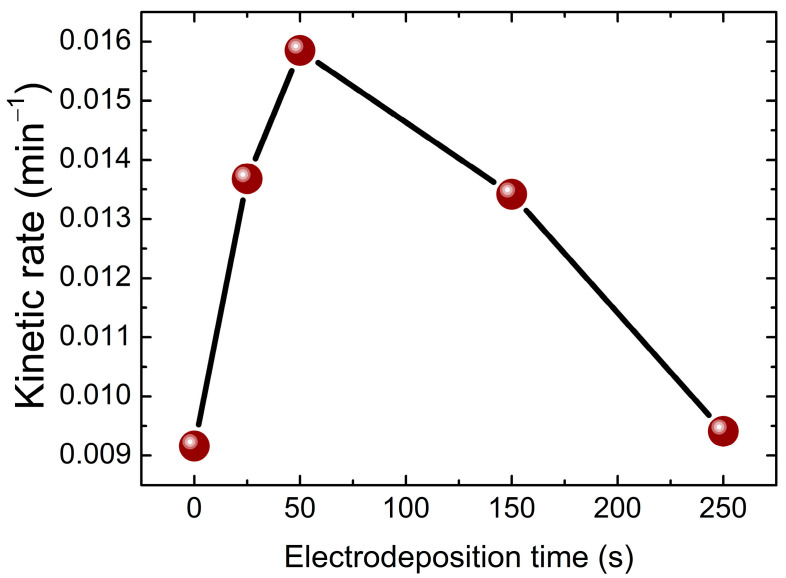
Kinetic constant of bare TiO_2_ and BiVO_4_/TiO_2_ nanocomposites with different electrodeposition time 25 s, 50 s, 150 s, 250 s for Amido Black degradation reaction.

The rate of the photodegradation kinetic was investigated with the given equation below [56]:

(5) $$\ln\left(\frac{C_{0}}{C_{t}}\right)=kt$$

where C_0_ and C_t_ are the concentrations of the dye at time 0 and t, respectively, and k is the rate constant. The slope of $\ln\left(\frac{C_{0}}{C_{t}}\right)$ plots provide the kinetic of photodegradation reaction rate ‘k’, which is presented in Figure 12. Compared to the pure TiO_2_ nanotubes, all the samples deposited with BiVO_4_ nanoparticles exhibited higher kinetic rate constant (k), especially the 50 s sample with k = 0.0159 min^−1^, which corresponds to the maximum removal percentage 99% and is in correlation with the optical and structural results where the 50 s sample had the largest crystallite size and lowest microstrain as well as the lowest band gap energy.

**Figure 13:**
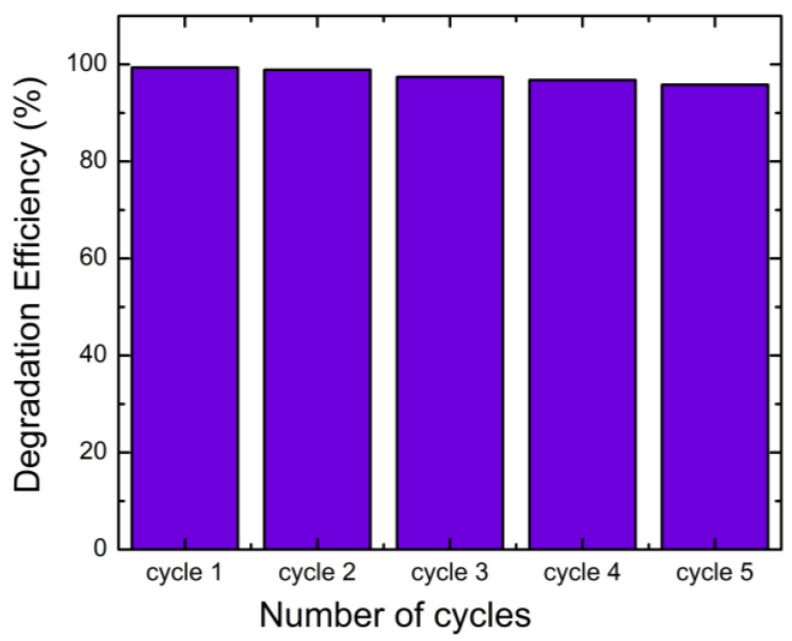
Recycle test for BiVO_4_/TiO_2_-50 s electrodeposition time for five consecutive cycles of photodegradation of AB.

The stability and the reusability of the sample was investigated as shown in Figure 13. The photocatalytic activity of BiVO_4_/TiO_2_ composites remains at a high capacity even after five consecutive photocatalytic performances for AB degradation. It only displays less than 4% deactivation after five cycles.

### 2.5. Quenching Test

**Figure 14:**
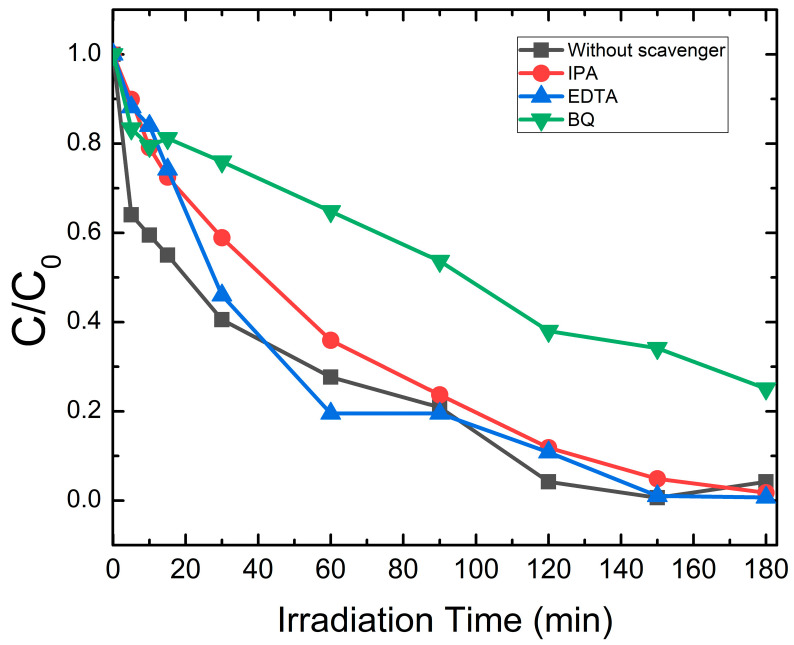
Photocatalytic evaluation of AB degradation for BiVO_4_/TiO_2_-50 s electrodeposition time with the presence of active species scavengers.

There are a variety of scavengers to use for the quenching of active oxygen species, including IPA, EDTA and BQ. These scavengers can trap holes (h^+^), hydroxyl radicals (OH^•^) and superoxide radicals (O_2_^•−^), respectively [57]. Figure 14 depicts the effect of scavengers on the degradation of AB under UV light for 180 min. The scavenger trapping test was conducted to better understand the photocatalytic mechanism and assess the effect of the responsible reactive molecules in the photocatalytic approach.

**Figure 15:**
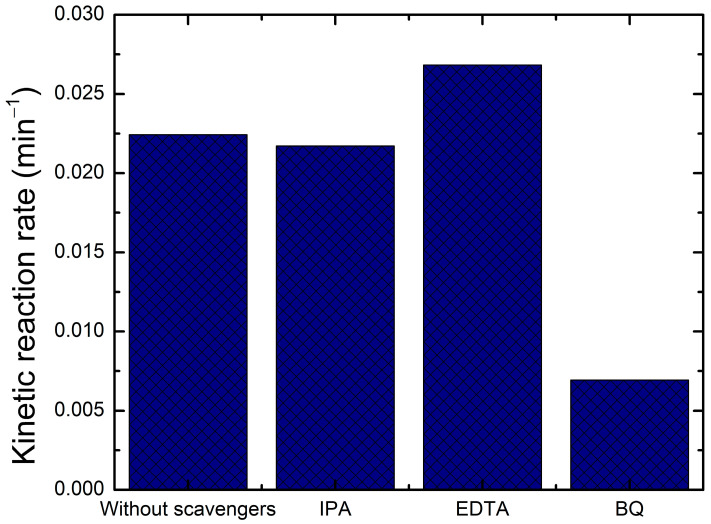
Photocatalytic reaction rate k of AB degradation for BiVO_4_/TiO_2_-50 s electrodeposition time with and without the presence of active species scavengers.

Figure 15 is a comparison of kinetic rate constant k for the BiVO_4_/TiO_2_-50 s electrodeposition time with and without scavengers; it seems that the kinetic rate intensely decreases with the addition of BQ, which means that the superoxide radicals (O_2_^•−^)are the ones responsible for the degradation of Black Amido or precisely, the ones that are mostly generated in these reactions. The significant increase in k with the addition of EDTA could be explained by the capturing of holes, which increases the separation of electron/hole pairs, thus, decreasing the recombination rate and the excessive existence of electrons favor the generation of superoxide radicals (O_2_^•−^), which were previously proven to be responsible for the degradation of AB. However, with the addition of IPA, the constant rate vaguely decreases, indicating that hydroxyl radicals (OH^•^) are not as important in the photodegradation of AB. These results are in accordance with the published literature [58], where the only vague decrease in the rate constant upon the addition of IPA (a scavenger of OH^•^ radicals) provides crucial evidence that a classical OH^•^ mediated oxidation pathway is minor. This is a key signature of the proposed Z-scheme mechanism. In this system, the photogenerated holes in the TiO_2_ valence band, which are the typical source of OH^•^ radicals, are predominantly consumed by internal recombination with electrons from the BiVO_4_ conduction band. Consequently, the generation of OH^•^ is suppressed. The primary oxidative power is instead provided by the holes accumulated in the BiVO_4_ valence band, which can directly oxidize the pollutant, alongside the dominant action of superoxide radicals (O_2_^•−^).

### 2.6. Mechanism of Photocatalysis

The synthesization of p-n heterojunction BiVO_4_/TiO_2_ and the position of band edges after the alignment is of great interest for the amelioration of photocatalytic performances. Under irradiation, both photocatalysts photogenerate electron/hole pairs that migrate to the surface of the material p-n heterojunction, where the electrons will react with oxygen molecules for the development of superoxide radicals (O_2_^•−^)and the holes will react with water molecules for the development of hydroxyl radicals OH^•^, as demonstrated in the following equations [59]

TiO_2_ + hν → e^−^ (CB) + h^+^ (VB) (6)

O_2_ + e^−^ → (O_2_^•−^) (7)

h^+^ + H_2_O → OH^•^ + H^+^ (8)

h^+^ + OH^−^ → OH^•^ (9)

**Figure 16:**
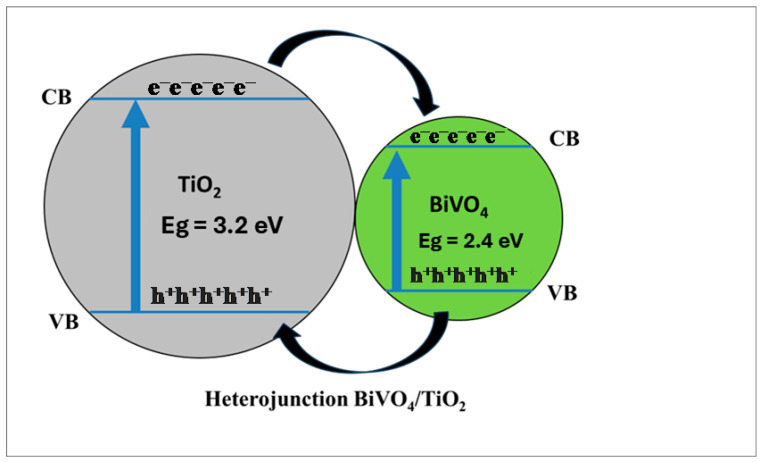
Charge carrier transfer and separation mechanism for BiVO_4_/TiO_2_ nanocomposite.

The same mechanism is performed on the surface of both photocatalysts, and after the production of these reactive species, ROS, they engage in the degradation of organic pollutants present in wastewater. The separation phenomenon of the photogenerated charge carries effectively reduces the recombination rate and enhances the photocatalytic efficiency of BiVO_4_/TiO_2_ nanocomposites; according to the literature, BiVO_4_ nanoparticles exhibit a work function of χ = 6.04 eV, while TiO_2_ possesses a χ = 5.8 eV [58] after contact and the band alignment, as shown in Figure 16, is where the electrons transfer from the conduction band of TiO_2_ to BiVO_4_ and the holes migrate from the valence band of BiVO_4_ to TiO_2_.

## 3. Discussion

The method used demonstrates that electrodeposition of BiVO_4_ on TiO_2_ nanotubes is an effective and simple method for enhancing photocatalytic activity. The fabrication of BiVO_4_ nanostructures was successful via a two-step process: electrodeposition of Bi nanoparticles, followed by heat treatment. The Bi deposition time had a significant impact on the composite properties. SEM analysis revealed that with increasing deposition time (from 25 s to 250 s), the morphology of the deposited BiVO_4_ changed from irregular and dispersed nanosheets (25 s, 50 s) to larger and aggregated structures, forming a porous film partially covering the TiO_2_ nanotubes at longer times (150 s, 250 s).

The photocatalytic performance for the degradation of Amido Black was highly dependent on the electrodeposition time. The BiVO_4_/TiO_2_ sample with a deposition time of 50 s exhibited the best degradation efficiency (99.4% in 150 min) and the fastest reaction kinetics. This optimal performance is attributed to a synergistic combination of structural and optical properties. XRD analysis indicated that the TiO_2_ (101) crystallites in the 50 s sample possessed the largest size (43.74 nm) and the lowest microstrain, suggesting that high crystallinity is favorable for charge transport. Although the size of the BiVO_4_ crystallites themselves is smaller at short deposition times, their presence and optimal distribution, confirmed by TEM and EDX, are crucial. The BiVO_4_ nanoparticles act as a co-catalyst, forming a heterojunction with TiO_2_ that promotes the separation of photogenerated electron–hole pairs. This is supported by the photoluminescence (PL) spectra, where the 50 s sample exhibited lower PL intensity, indicating reduced charge carrier recombination.

The optical properties were also optimized for the 50 s deposition time, as this sample exhibited the lowest optical band gap (2.6 eV). The heterojunction between BiVO_4_ and TiO_2_ facilitates a Z-type charge transfer mechanism, where electrons from the TiO_2_ conduction band combine with holes from the BiVO_4_ valence band. This process effectively separates the strongest reducing and oxidizing agents, increasing the generation of reactive oxygen species (ROS). Trapping tests confirmed that superoxide radicals (O_2_^•−^) are the main active species in the degradation process. Furthermore, the BiVO_4_/TiO_2_-50 s composite demonstrated excellent stability, maintaining high photocatalytic activity over five consecutive cycles with minimal deactivation (<4%). In conclusion, the BiVO_4_/TiO_2_ heterojunction, particularly with an optimized electrodeposition time of 50 s, represents a promising and effective catalyst for water remediation and pollutant degradation applications. Moreover, the performance of BiVO_4_/TiO_2_ in AB degradation was found to be better or comparable to those of the similar tabular forms of catalysts reported in the recent literature, as summarized in Table 5.

## 4. Materials and Methods

### 4.1. Chemicals

The reagents used in this work are bismuth (III) nitrate pentahydrate (Bi(NO_3_)_3_.5H_2_O, 99.99%), lithium perchlorate (LiClO_4_, 99%), dimethyl sulfoxide (DMSO, extra pure grade), vanadyl acetylacetonate (VO (acac)_2_, 99%), and sodium hydroxide (NaOH, extra pure grade). All these chemicals were purchased from Sigma-Aldrich (St. Louis, MO, USA).

### 4.2. Synthesis of TiO_2_ Nanotubes (NTs)

A second-grade Ti foil with a dimension of 2.5 cm × 2.5 cm × 1 mm (99.7%) was used. The surface of the foil was first polished with sanding papers of grades ranging from 320 to 2200. Then, it was ultrasonically cleaned for 10 min, with ethanol, acetone, and double-distilled water, in that order. The elaboration of TiO_2_ NTs from Ti substrates involved a two-step anodization: 45 min for the first anodization and 120 min for the second one under a fixed voltage of 60 V at room temperature. The process was performed in an electrolytic cell containing 100 mL of ethylene glycol + 1 wt% NH_4_F + 2 v% H_2_O. To ensure the anatase crystal phase of TiO_2_ was obtained, the samples were annealed in an oven at 450 °C for 3 h.

### 4.3. Synthesis of BiVO_4_ Nanoparticles on TiO_2_-NTs

Two steps are required for the electrodeposition of BiVO_4_ on titanium dioxide NTs. Firstly, the Bi thin films were prepared by the electrodeposition method for four periods of time: 25, 50, 150 and 250 s. We used an electrolyte bath containing 50 mL of dimethyl sulfoxide (DMSO), 20 mM of Bi(NO_3_)_3_.5H_2_O, and 0.1 M LiClO_4_. A three-electrode Autolab PGSTAT30 potentiostat/galvanostat PGSTAT30 was used for the electrodeposition process: titanium NTs substrate as working electrode, Ag/AgCl (3 M NaCl) as reference electrode, and Pt wire as counter electrode. The temperature of the plating solution was fixed at 60 °C and the optimized cathodic potential was −1.6 V (vs. Ag/AgCl). After each electrodeposition, the dark-colored Bi films were carefully immersed in DMSO solution for 3 min and dried in air at 70 °C for 1 h. Then, in order to convert Bi to BiVO_4_, a DMSO solution containing 50 μL of 50 mM VO (acac)_2_ was spread over the entire surface of the Bi film by dip coating and completely immersing the electrode in the solution. The film was then heated at 450 °C for 3 h in air (ramping rate = 2 °C min^−1^). During the heating process, Bi-metal and VO^2+^ species were oxidized to Bi_2_O_3_ and V_2_O_5_, respectively. Then, they reacted with each other to form BiVO_4_. The VO^2+^ ions were in excess to ensure the complete conversion of Bi to BiVO_4_. This excess led to the formation of yellow-green residual V_2_O_5_ layer that could be easily removed by soaking the samples in 1 M NaOH solution for 30 min while stirring. BiVO_4_ samples were obtained for four Bi deposition times (25, 50, 150, and 250 s).

### 4.4. Samples Characterization

In order to examine the morphology of the prepared samples, we used a 15 kV Scanning Electron Microscope (SEM, FEI XL30 ESEM, Hatfield, PA, USA) and Transmission Electron Microscopy (TEM, FEI Tecnai G2) equipped with X-ray energy dispersive analysis (EDX) operating at 200 kV. For structural study, X-ray diffraction was performed using Philips X’ PERTMPD diffractometer (Amsterdam, The Netherlands), which uses CuKα radiation (λ = 1.5406 Å). The diffraction patterns ranged in 2θ = 10–80°. The spectra were collected with a Thermo Scientific K-Alpha X-Ray Photoelectron Spectrometer (XPS, Waltham, MA, USA) System using monochromatized Al Kα (1486.6 eV). Optical properties of pure TiO_2_ NTs and BiVO_4_-TiO_2_NTs were investigated by UV–Vis-NIR photoluminescence (PL) using a Perkin Elmer Lambda 950 spectrophotometer (Waltham, MA, USA) between 200 and 1200 nm and Perkin Elmer LS55 coupled to xenon lamp at an excitation wavelength of λ = 340 nm.

### 4.5. Photocatalytic Activity Measurements

#### 4.5.1. Photodegradation of Black Amido

The photocatalytic activity of the prepared samples (bare TiO_2_ NTs and BiVO_4_/TiO_2_ nanocomposites) was evaluated by monitoring the degradation of Amido Black 10B (AB) dye under UV light irradiation. The process was conducted at ambient temperature (~25 °C).

Aqueous solutions of AB dye with a concentration of 5 mg·L^−1^ were prepared. For each test, approximately 10 mL of the dye solution was placed in a Petri dish, and the photocatalyst sample (a thin film on a Ti foil substrate with dimensions of 2.5 cm × 1 cm) was immersed into the solution.

Prior to irradiation, the system was kept in complete darkness under constant magnetic stirring for 15 min to establish an adsorption–desorption equilibrium between the dye molecules and the catalyst surface. This step ensures that the subsequent degradation was due to photocatalytic reactions and not merely physical adsorption.

After the dark adsorption period, the solution was irradiated using a 15 W OSRAM germicidal UV lamp with a primary emission wavelength of λ = 256 nm. The lamp, with a length of 45 cm, was positioned at a fixed distance above the Petri dishes to ensure uniform illumination. Appropriate safety measures (UV-protective glasses and gloves) were strictly followed during irradiation.

At predetermined time intervals (5, 10, 15, 30, 45, 60, 90, 120, 150, 180, 210, 240, and 270 min), small aliquots (~1 mL) of the solution were extracted from the Petri dish. The concentration of AB dye in the collected samples was monitored by measuring the absorbance at its characteristic maximum wavelength (λ_max_ = 618 nm) using a UV–Vis spectrophotometer. The degradation efficiency was calculated based on the decrease in absorbance relative to the initial concentration after the dark adsorption step.

#### 4.5.2. Trapping Test

To better understand which of the generated ROS are responsible for the massive degradation of Black Amido with the photocatalysts BiVO_4_/TiO_2_, a collective group of scavengers have been used, such as EDTA, IPA and BQ; each is assigned to capture a species from the ROS hole h^+^, radical’s hydroxyl OH^•^ and radical’s superoxide (O_2_^•−^). The equations below explain the quenching processes [63,64]:

- Quenching of Hydroxyl Radicals (OH^•^) by Isopropanol (IPA)
  Isopropanol reacts with hydroxyl radicals via hydrogen abstraction, leading to the formation of acetone and water.

Chemical Equation:

(CH_3_)_2_CH-OH + OH^•^ → (CH_3_)_2_C-OH + H_2_O

2^•^ (CH_3_)_2_C-OH → (CH_3_)_2_C(OH)-C(OH)(CH_2_)_2_ (Dimerization)

^•^(CH_3_)_2_C-OH + O_2_ → (CH_3_)_2_C=O + HO_2_^•^ (Formation of Acetone and a hydroperoxyl radical)

▪ Quenching of Superoxide Radical Anions (O_2_^•−^) by Benzoquinone (BQ)
  Benzoquinone (BQ) readily accepts an electron from the superoxide radical, forming a semiquinone radical and then hydroquinone after protonation.

Chemical Equation:

O_2_^•−^ + BQ → O_2_ + BQ^•−^ (Semiquinone Radical Anion)

BQ^•−^ + H^+^ → HBQ^•^ (Semiquinone Radical)

HBQ^•^ + O_2_^•−^ + H^+^ → H_2_BQ (Hydroquinone) + O_2_

▪ Quenching of Photogenerated Holes (h^+^) by Ethylenediaminetetraacetic Acid (EDTA)
  EDTA acts as a sacrificial electron donor, capturing the photogenerated holes before they can generate OH^•^ radicals or directly oxidize the dye.

Chemical Equation (Simplified):

EDTA + h^+^ → EDTA^•+^ (Oxidized EDTA)

## 5. Conclusions

In summary, this study demonstrates that electrodeposition of BiVO_4_ on TiO_2_ nanotubes is an effective and simple method for enhancing photocatalytic activity. The fabrication of BiVO_4_ nanostructures was successful via a two-step process: electrodeposition of Bi nanoparticles followed by heat treatment. The Bi deposition time had a significant impact on the composite properties. SEM analysis revealed that with increasing deposition time (from 25 s to 250 s), the morphology of the deposited BiVO_4_ changed from irregular and dispersed nanosheets (25 s, 50 s) to larger and aggregated structures, forming porous nanoflower-like structures partially covering the TiO_2_ nanotubes at longer times (150 s, 250 s).

The photocatalytic activity of the BiVO_4_/TiO_2_ composites was found to be strongly influenced by the electrodeposition time, with the sample deposited for 50 s exhibiting the highest degradation efficiency toward Amido Black (99.4% within 150 min). This outstanding performance results from a well-balanced combination of structural and optical characteristics. XRD analysis revealed that the TiO_2_ (101) crystallites in the 50 s sample possessed the largest size and lowest microstrain, reflecting enhanced crystallinity and improved charge carrier transport. Meanwhile, TEM and EDX analyses confirmed the uniform dispersion of BiVO_4_ nanoparticles, which promotes the formation of a well-defined BiVO_4_/TiO_2_ heterojunction. Such intimate interfacial contact effectively facilitates charge separation, as further evidenced by the reduced PL emission intensity.

In addition, the narrowed optical band gap (2.6 eV) of the BiVO_4_/TiO_2_-50 s composite enhances visible-light absorption and contributes to superior photocatalytic efficiency. The Z-scheme charge transfer mechanism established between BiVO_4_ and TiO_2_ maintains strong redox potentials, favoring the formation of reactive oxygen species—primarily superoxide radicals (O_2_•−) as confirmed by radical-trapping experiments. Furthermore, the BiVO_4_/TiO_2_-50s composite displayed remarkable cycling stability, preserving over 96% of its initial performance after five consecutive runs. Overall, the optimized BiVO_4_/TiO_2_ heterojunction with a 50 s deposition time demonstrates high efficiency, robustness, and durability, highlighting its great promise as a sustainable photocatalyst for wastewater treatment and environmental remediation.

## Figures and Tables

**Table 1:** Comparison of catalysts/TiO_2_ for the photodegradation of AB.

Catalyst	Conditions	Removal Efficiency (%)	Reaction Time (min)	Ref.
Zn/Mg co-doped TiO_2_ (ZMT4)	[AB] = 10 mg.L^−1^ [Catalyst] = 100 mg.L^−1^	99%	20	[1]
PNA/TiO_2_	[AB] = 100 mg.L^−1^ [Catalyst] = 200 mg	90%	60	[42]
Co/TiO_2_	[AB] = 100 μg.L^−1^ [Catalyst] = 1.0 g.L^−1^	90%	60	[43]
BiVO_4_/TiO_2_-NTs	[AB] = 5 mg.L^−1^ [Catalyst] = thin film	99.4%	150	This work

**Table 2:** Crystallite size and microstrain of BiVO_4_/TiO_2_ for the preferred orientation TiO_2_ (101), BiVO_4_ (004) and Bi_2_O_3_ (111) at different electrodeposition times.

Samples	TiO_2_ (101)		BiVO_4_ (004)		Bi_2_O_3_ (111)	
	Crystallite Size	Microstrain (×10^−3^%)	Crystallite Size	Microstrain (×10^−3^%)	Crystallite Size	Microstrain (×10^−3^%)
0 s	41.09	3.82	-	-	-	-
25 s	42.45	3.76	-	-	-	-
50 s	43.74	3.65	-	-	-	-
150 s	35.65	4.48	37.9	3.33	28.16	5.99
250 s	37.92	4.19	42.1	2.99	41.31	4.07

**Table 3:** Weight % of the chemical composition of BiVO_4_/TiO_2_ at 250 s electrodeposition time.

Element	Weight%	Atom%	Formula	Compound%
O	40.69	64.94	O	40.69
Na	8.89	9.88	Na	8.89
Ti	45.56	24.29	Ti	45.56
V	0.79	0.39	V	0.79
Bi	4.08	0.50	Bi	4.08
Total	100.00	100.00		100.00

**Table 4:** Band gap energy and crystallite size of bare TiO_2_ and BiVO_4_/TiO_2_ nanotubes with different electrodeposition times: 25 s, 50 s, 150 s, 250 s.

Samples	Crystallite Size [TiO_2_ (101)] (nm)	Optical Band Energy (eV)
TiO_2_	41.09	3.3
BiVO_4_/TiO_2_-25 s	42.45	2.61
BiVO_4_/TiO_2_-50 s	43.74	2.6
BiVO_4_/TiO_2_-150 s	35.64	2.66
BiVO_4_/TiO_2_-250 s	37.92	2.7

**Table 5:** Comparative work of BiVO_4_/TiO_2_ under different photocatalytic conditions.

Catalyst	Conditions	Photodegradation Efficiency (%)	Degradation Time (min)	Ref.
3DOM BiVO_4_/TiO_2_	Concentration of RhB = 10^−3^ M Catalyst mass = 20 mg	80	120	[60]
BiVO_4_/TiO_2_ nanocomposites	Concentration of AB113 = 40 mg.L^−1^ Catalyst loading = 1 g.L^−1^	82	120	[61]
BiVO_4_/TiO_2_ composite	Concentration of MB = 2 × 10^−5^ M Catalyst loading = 1 g.L^−1^	84	120	[62]
BiVO_4_/TiO_2_-NTs	Concentration of AB = 5 mg.L^−1^ Catalyst = thin film	99.4	150	This work

## Data Availability

The original contributions presented in this study are included in the article. Further inquiries can be directed to the corresponding authors.
